# *Hymenolepis nana* Infection in an HIV Patient With Diarrhea

**DOI:** 10.14309/crj.0000000000000709

**Published:** 2021-11-24

**Authors:** Roohi Patel, Peter Dellatore, Colton Smith, Anish Vinit Patel

**Affiliations:** 1 Department of Medicine, Rutgers Robert Wood Johnson Medical School, New Brunswick, NJ; 2 Division of Gastroenterology and Hepatology, Rutgers Robert Wood Johnson Medical School, New Brunswick, NJ; 3 Department of Pathology and Laboratory Medicine, Rutgers Robert Wood Johnson Medical School, New Brunswick, NJ

## CASE REPORT

**Figure 1.**
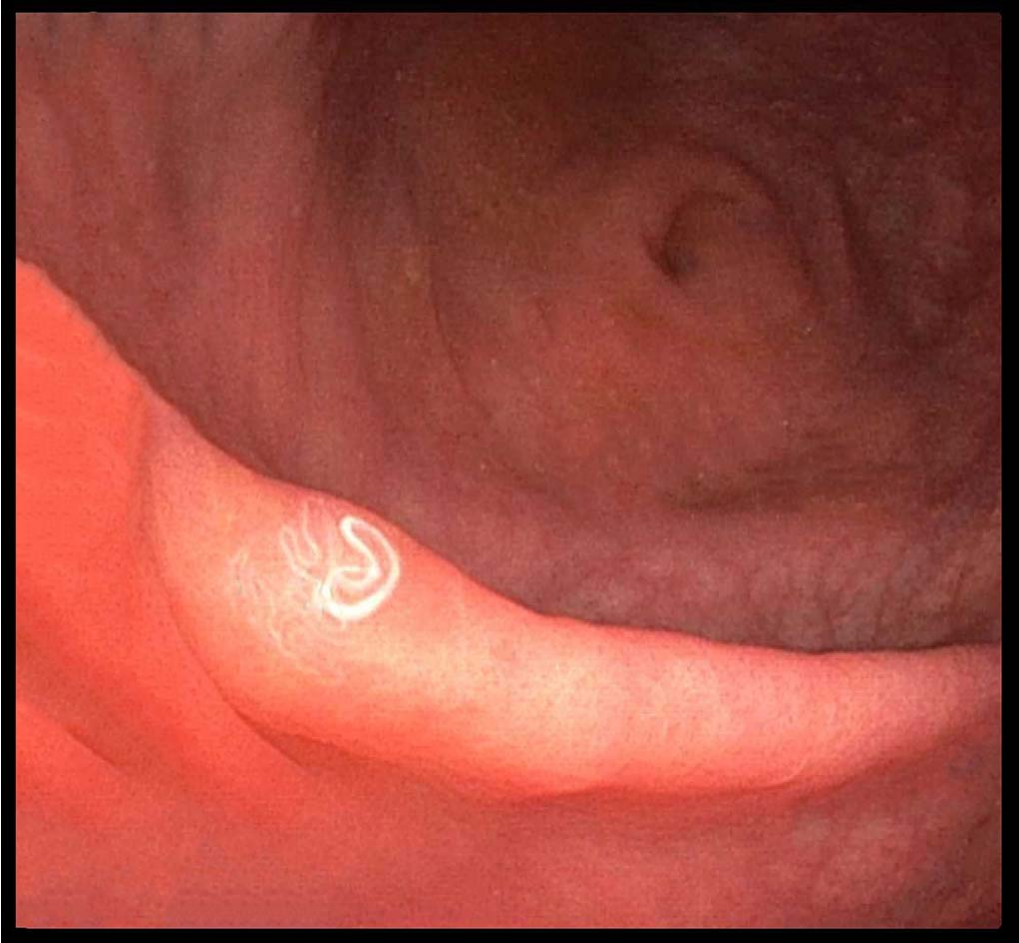
Colonoscopy showed multiple linear and coiled white thread-like worms on the mucosa of the terminal ileum and cecum. This worm was removed using forceps for diagnosis.

**Figure 2.**
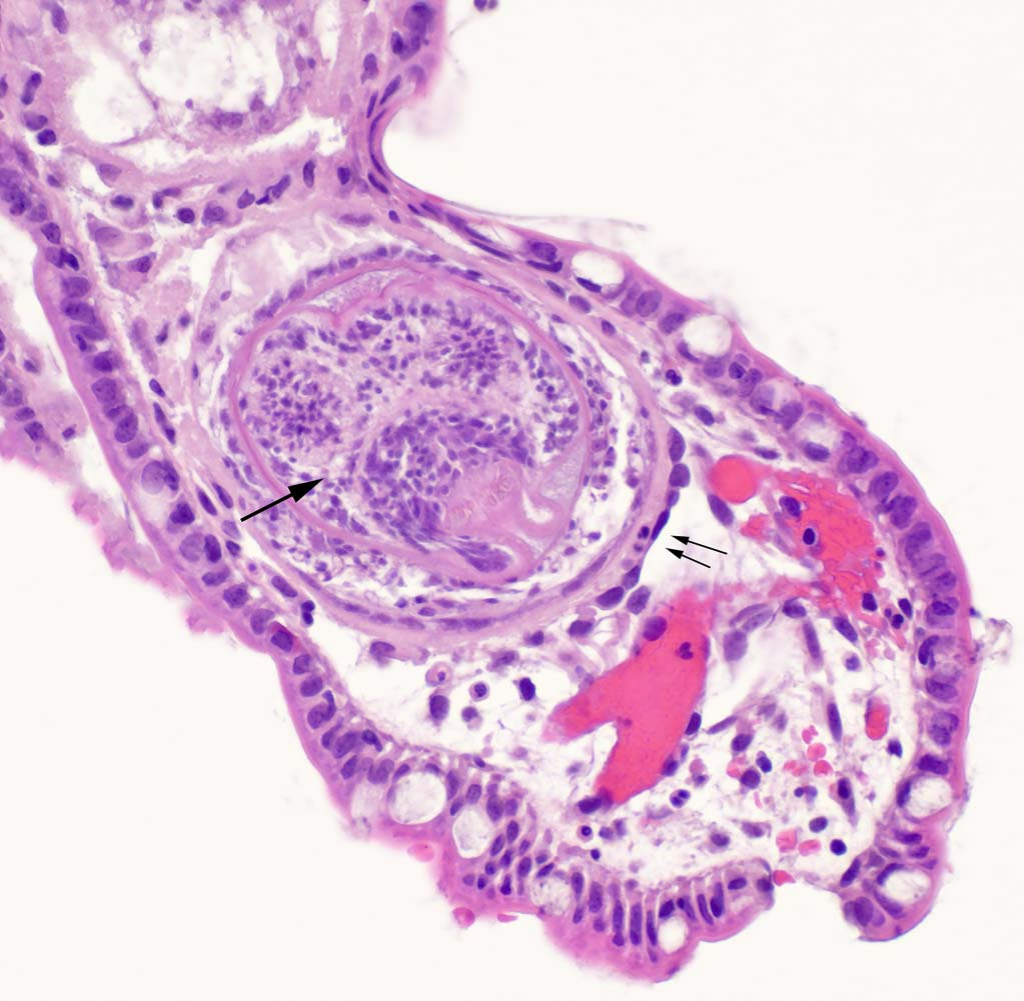
Biopsy of the terminal ileum revealed multiple parasitic organisms. One organism is visualized with double arrows showing the outer membrane and a single arrow showing the hexacanth embryo or oncosphere.

**Figure 3.**
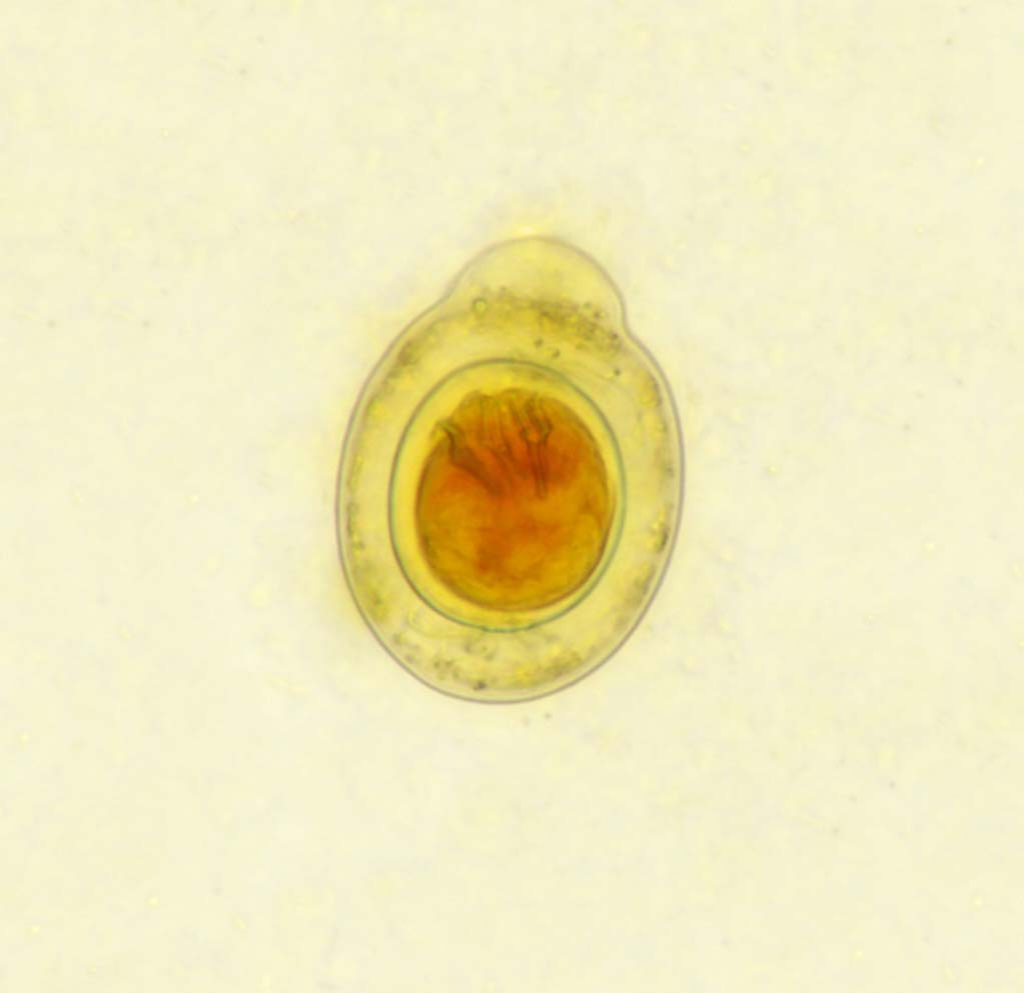
Stool examination showed ova consistent of *Hymenolepis nana.*

A 27-year-old man with untreated human immunodeficiency virus and acquired immunodeficiency syndrome presented with 5 days of diffuse abdominal pain, watery, nonbloody diarrhea, vomiting, and a CD4 count of 93 cells/mm^3^. He endorsed 4–5 episodes of diarrhea per day along with nocturnal episodes. He had a recent diagnosis of cerebral toxoplasmosis and was undergoing treatment with trimethoprim-sulfamethoxazole and high-dose dexamethasone for 4 weeks. Abdominal computed tomography demonstrated normal bowel appearance. Stool studies were negative for acid-fast bacilli, *Salmonella*, *Shigella*, *Campylobacter*, *Yersinia*, *Vibrio*, and *Clostridium difficile* infection. Colonoscopy was performed to evaluate for *Cytomegalovirus* infection. He was found to have linear and coiled white thread-like worms on the mucosa of the terminal ileum and cecum (Figure 1). Biopsy of the terminal ileum revealed multiple parasitic organisms (Figure 2). The diagnosis was confirmed with stool examination that revealed ova consistent of *Hymenolepis nana* (*H. nana)* (Figure 3). The patient likely had a latent *H. nana* infection that was reactivated by high-dose steroid therapy. He received treatment with praziquantel, and his symptoms rapidly improved.

*H. nana* (also called dwarf tapeworm) is the most common human cestode infection, with an estimated 50 – 75 million carriers worldwide. This tapeworm is endemic to Asia, Africa, and Southern and Eastern Europe. Humans or rodents serve as the definitive host in its life cycle, whereas arthropods serve as the intermediate host. Humans are infected on ingestion of infected arthropods or embryonated eggs from contaminated food or water. After ingestion, the eggs hatch to release a 6-hooked larva that then penetrates the small intestine and develops into a cysticercoid larva. Autoinfection results in persistence of tapeworm and larvae for years if untreated. The infection is often asymptomatic; however, it can cause diarrhea when there is heavy worm burden or in immunosuppressed states.^1,2^ Careful endoscopic examination of the colon and small bowel with stool evaluation can aid in diagnosis of such gastrointestinal parasitic infections in immunocompromised individuals.

## DISCLOSURES

Author contributions: R. Patel, P. Dellatore, C. Smith, and A.V. Patel conceptualized, designed, drafted, revised, and approved the manuscript. A.V. Patel is the article guarantor.

Financial disclosure: None to report.

Informed consent was obtained for this case report.
